# Acceptability and satisfaction of project MOVE: A pragmatic feasibility trial aimed at increasing physical activity in female breast cancer survivors

**DOI:** 10.1002/pon.4662

**Published:** 2018-03-01

**Authors:** Tanya Pullen, Paul Sharp, Joan L. Bottorff, Catherine M. Sabiston, Kristin L. Campbell, Susan L. Ellard, Carolyn Gotay, Kayla Fitzpatrick, Cristina M. Caperchione

**Affiliations:** ^1^ School of Health and Exercise Sciences University of British Columbia Kelowna British Columbia Canada; ^2^ Institute for Healthy Living and Chronic Disease Prevention University of British Columbia Kelowna British Columbia Canada; ^3^ Faculty of Physical Education University of Toronto Toronto Ontario Canada; ^4^ Department of Physical Therapy University of British Columbia Vancouver British Columbia Canada; ^5^ Cancer Centre of the Southern Interior British Columbia Cancer Agency Kelowna British Columbia Canada; ^6^ School of Population and Public Health University of British Columbia Vancouver British Columbia Canada

**Keywords:** cancer, community‐based intervention, financial incentives, health promotion, microgrants, oncology, physical activity, survivorship, women

## Abstract

**Objective:**

Despite the physical and psychological health benefits associated with physical activity (PA) for breast cancer (BC) survivors, up to 70% of female BC survivors are not meeting minimum recommended PA guidelines. The objective of this study was to evaluate acceptability and satisfaction with Project MOVE, an innovative approach to increase PA among BC survivors through the combination of microgrants and financial incentives.

**Methods:**

A mixed‐methods design was used. Participants were BC survivors and support individuals with a mean age of 58.5 years. At 6‐month follow‐up, participants completed a program evaluation questionnaire (n = 72) and participated in focus groups (n = 52) to explore their experience with Project MOVE.

**Results:**

Participants reported that they were satisfied with Project MOVE (86.6%) and that the program was appropriate for BC survivors (96.3%). Four main themes emerged from focus groups: (1) acceptability and satisfaction of Project MOVE, detailing the value of the model in developing tailored group‐base PA programs; (2) the importance of Project MOVE leaders, highlighting the value of a leader that was organized and a good communicator; (3) breaking down barriers with Project MOVE, describing how the program helped to address common BC related barriers; and (4) motivation to MOVE, outlining how the microgrants enabled survivors to be active, while the financial incentive motivated them to increase and maintain their PA.

**Conclusion:**

The findings provide support for the acceptability of Project MOVE as a strategy for increasing PA among BC survivors.

## 1 BACKGROUND

Breast cancer (BC) is the most common cancer among women worldwide.^1^ Following surgery and treatment, many BC survivors experience negative short‐term and long‐term side effects that span physical and mental health.^2^, ^3^, ^4^ Physical activity (PA) is an effective non‐pharmaceutical intervention strategy that can help improve many of these side effects.^5^, ^6^, ^7^ Moreover, PA has been associated with numerous health benefits among cancer survivors, including weight management, reduced pain and fatigue, reduced depression and anxiety, and reduced mortality and BC reoccurrence.^8^, ^9^, ^10^ In spite of the benefits associated with being physically active, up to 70% of BC survivors are not meeting the minimum recommended guidelines of 150 minutes of moderate to vigorous PA per week.^11^, ^12^, ^13^ While PA programs designed for BC survivors have shown potential in increasing engagement,^14^, ^15^, ^16^ many of these programs do not meet the specific needs of BC survivors.^17^, ^18^ One promising approach to address this gap is the combined use of microgrants and financial incentives.

The microgrant model refers to a scheme in which small amounts of funds are awarded to successful community‐based applicant groups to develop and/or implement a community program or initiative. Although relatively unique to the health promotion field, a small number of studies have shown that these schemes can stimulate community health‐related activities, help build confidence to undertake and engage in health promoting behaviors, and provide an outlet for social interaction.^19^, ^20^, ^21^, ^22^, ^23^ It has also been reported that similar microgrant schemes have aided in improving PA and healthy eating behaviors in priority communities, such as ethnic minorities and low socioeconomic groups.^21^, ^23^ The microgrant model has been framed within social ecological models of behavior change^24^ whereby environmental, social, and individual factors may be impacted for enacted change in behavior. Aligned with social cognitive theory,^25^ financial incentives within the microgrant model also provide feedback and reinforcement of behavior change success that may enhance self‐efficacy and sustained behavior change.^26^ Within this context, Project MOVE^27^ was created to prompt and sustain PA among BC survivors by combining the use of microgrants and financial incentives. This unique combination was used to promote PA in the context of BC by returning some of the decision‐making power back to BC survivors and instilling a sense of control over their health. Thus, the purpose of this study was to examine the feasibility of this model by gaining a greater understanding of the acceptability and satisfaction with Project MOVE.

## 2 METHODS

### 2.1 Participants and recruitment

Participants were recruited as pre‐existing or newly formed groups of 8 to 12 adult (18+ years) female BC survivors living in the Okanagan region of British Columbia, Canada. Individual women who were not able to form a group independently were asked to contact the research team who facilitated connections to join an existing group or lead a group. Support women (eg, sisters, friends) were allowed to participate in the groups, with a caveat that the groups composed of at least 50% BC survivors. A variety of recruitment methods were utilized including face‐to‐face meetings with community stakeholders (eg, Canadian Cancer Society [CCS], BC Cancer Agency), attendance at relevant community events (eg, Run for the Cure), and advertisements through local print, radio media, and social media (eg, Facebook, Twitter).

To address the purpose of the current study, participants included women who were part of an existing Project MOVE group and attended the 6‐month follow‐up data collection session. All participants completed written informed consent at baseline, and verbal consent was renewed prior to each focus group session. Ethical approval was obtained from the University of British Columbia's Behavioral Research Ethics Board (#H14‐02502).

### 2.2 Project MOVE intervention

A full description of the Project MOVE intervention has been previously reported.^27^ In brief, BC survivors were encouraged to come together as a group to develop and implement their own PA initiative based on their needs and preferences, and more importantly, to address any unique circumstances and specific barriers that may have limited them from being active. Groups were then invited to apply for a microgrant of up to $2000 to support these initiatives. Once submitted, all grants were reviewed by a grant review panel, consisting of members from the research team, representatives from CCS, and the target population. Upon recommendation from the panel, microgrant funds were distributed to the successful applicant groups (Table 1). Unsuccessful applicant groups (*n* = 5 groups) were provided with feedback and encouraged to revise and re‐submit their application for re‐review. Participant groups were also informed that if they increased the group's combined PA (assessed at 6 months), they would be awarded a further $500 to support more PA sessions or a group social event (see supplemental Appendix 1). Each group included a leader who was responsible for communicating with the research team, organizing participants, and coordinating activities.

**Table 1:** Project MOVE groups

Applicant Groups	Participants at Baseline	Utilization of Microgrant
Round 1		
1) women on weights	5	Exercise trainer
2) group training	10	Exercise trainer
3) explore movement	8	Exercise trainer
4) move anytime anywhere	12	Exercise trainer
5) strive to thrive	12	Fitbits™ and weights
Round 2		
6) spin together	8	Spin class passes
7) fit together	12	Exercise trainer
8) new wave warriors	9	Exercise trainer
9) iHealth	7	Exercise trainer
10) spin to health	6	Spin, barre, yoga passes

### 2.3 Measures and procedures

The 6‐month follow‐up consisted of a brief self‐report program evaluation questionnaire and participation in a focus group.

#### 2.3.1 Program evaluation questionnaire

Participants (*n* = 72) were asked to rate their Project MOVE experience on a 5‐point Likert scale, with 1 representing “strongly disagree” and 5 representing “strongly agree.” The questionnaire included a total of 5 questions related to program satisfaction, acceptability, and appropriateness.

#### 2.3.2 Focus groups

Participants' experiences with the program were explored with focus group methodology. One focus group was held for each Project MOVE group with a total of 52 participants (groups ranging 3‐7 participants). Each focus group was held at a time and location convenient for participants, lasted 35 to 60 minutes, and was audio‐recorded using a digital SonyTM recorder (ICD‐PX333). Semi‐structured open‐ended questions and prompts were used to guide the discussion (see supplemental Appendix 2),^28^ and sticky notes, flip charts, and pens^29^ were also available to generate involvement from participants. Similarly, non‐BC women who were part of the groups were part of the focus groups to encourage discussion. Any written material that was not captured verbally in discussion and perceptions from non‐BC survivors within the groups were not included in the analysis.

### 2.4 Analysis

The responses from the program evaluation questionnaire were reported as percentages. Focus group discussions were transcribed verbatim, and all identifiable information was removed to ensure anonymity and confidentiality following transcription. A thematic content analysis^30^ was conducted to explore participants' experiences with Project MOVE. All data were independently coded and categorized by 2 research team members using NVivo11™. Each team member systematically read the transcripts multiple times, highlighted segments of interest, identified, and coded potential themes. Once coding was complete, themes were discussed among the 2 researchers to ensure bias was minimized. Any discrepancies that arose during analysis were presented and discussed further until a consensus was reached.

## 3 RESULTS

### 3.1 Sample characteristics

Baseline demographic data were collected for a total of 87 participants (see supplemental Appendix 3). At 6‐month follow‐up, data were collected for a total of 72 participants (15 non BC survivors). There were no statistical differences between those who completed the follow‐up assessment versus those who dropped out (see supplemental Appendix 1) between baseline and 6 months. Of the 72 participants (15 non BC survivors) that completed the 6‐month follow‐up, 52 participants (11 non BC survivors) also participated in the focus groups. Those that completed the 6‐month follow‐up (*n* = 72) were primarily BC survivors (79%), white (95%), and married (68%), with a mean age of 58.5 ± 8.8 years. Mean group PA increased by 990 steps per day from baseline to 6‐month follow‐up.

### 3.2 Program evaluation results

Results from the program evaluation questionnaire represent responses from those identified as BC survivors (*n* = 57). These participants indicated that they were satisfied with Project MOVE (86.6%), they learnt new things about PA through Project MOVE (70.3%), Project MOVE was appropriate for female BC survivors (96.3%), they enjoyed being part of a Project MOVE group (94.5%), and would recommend the program to another BC survivor (94.5%).

### 3.3 Focus group results

Four themes emerged from the focus group data. Findings from each theme are summarized with representative quotes from participants who were BC survivors (*n* = 41).

#### 3.3.1 Acceptability and satisfaction of project MOVE

Participant comments reflected their satisfaction with Project MOVE and its acceptability for BC survivors. Participants emphasized the importance of the microgrant and financial incentive in facilitating PA; “*Project MOVE gave us that little bit of funding to put a group together, learn some skills and meet some people. That was very helpful”* (group 1, participant 3). Many also indicated that Project MOVE enabled the exploration of new activities. One participant explained; *“For me it was an introduction to things I never experienced before like yoga, circuit training and spin. So, it was really good”* (group 8, participant 5). While each Project MOVE group utilized the microgrant and financial incentive in unique ways, 7 groups used the funds to hire an exercise trainer. Participants in these groups found the trainers particularly valuable, attributing them to learning how to perform exercises safely and use the gym equipment properly. “*The instructor spent a lot of time on technique which was really good”* (group 1, participant 3) *and “They did show us how to use equipment as well…like if you wanted to make this harder”* (group 4, participant 4) were indicative of many of the participant responses.

Further, participants emphasized the importance of the group aspect, particularly the supportive environment within the groups. One participant explained; “*It was nice exercising with people who have gone through what you have gone through. The friendships we've built and just the support here. That was nice”* (group 5, participant 3). Participants appreciated how the Project MOVE groups were structured to bring BC survivors together to participate in an activity that was enjoyable and meaningful to the group, rather than discussing the hardships associated with BC and survivorship. The majority of participants indicated that their group did not primarily identify as a “traditional” BC survivor group, but rather focused on moving beyond their cancer diagnoses towards a fit and healthy future. One participant explained;

I didn't want to join a support cancer group and just talk about our cancer. But I thought we have a connection [in our Project MOVE group]. It doesn't mean that you have to talk about [cancer] all the time. But exercise is a good vehicle for making some extra connections (group 3, participant 3).

#### 3.3.2 The importance of Project MOVE leaders

Many groups indicated that having a leader who communicated regularly with the group was important. One group highlighted the impressive level of engagement from their leader: *“She really was invested. Like she went above and beyond for us. She really wanted us to succeed. And we did”* (group 8, participant 6). Many groups also valued having a leader who initiated discussion on how to utilize the funds but also provided group members with the opportunity to offer input and participate in the decision‐making process.

By contrast, groups with leaders who were not as engaged with their group reported less enjoyment, as described by 1 participant; “*It really felt at the start we had a leader. Like [the leader] was running our program. But then it just sort of felt like communication was sporadic. We would throw out ideas [about activities] but not necessarily get a response”* (group 10, participant 1).

#### 3.3.3 Breaking down barriers with Project MOVE

Many women spoke about the choice of activities and the close comfortable nature of the group in breaking down some of the common barriers to PA. For example, many participants indicated that poor body image, as a result of treatment (ie, body changes and scars from surgery), was a barrier to being physically active in a public setting:

You see all these fit and beautiful people at the gym and I'm still in the middle of my breast reconstruction, so that is a barrier to going out to the gym outside of this group. For a breast cancer survivor that wants to get out there and get active again, [the concern is] I have no boobs or I have one boob or you put your arms up and scars are showing (group 4, participant 7).

However, being part of a small PA group provided an opportunity to develop relationships with fellow survivors, which in turn helped women feel more comfortable talking about their surgeries and body image issues and participating in PA. One participant explained, “*Because it's a smaller group I feel more comfortable and connected. With the big classes at the rec centre it's different”* (group 1, participant 1).

Financial hardship was also reported as a barrier to getting active as many participants took time off work during and post‐treatment, and couldn't afford a gym membership or to hire a personal trainer following treatment. Additionally, many survivors reported that prior to the program, they were hesitant to invest their own money towards a new activity because they were concerned that they would join a program they would not enjoy or was too physically challenging, as explained by 1 participant;

I couldn't quite frankly go to a jazzercize... Because, if I had joined and paid them money and realized I couldn't do it… I couldn't do it. So, what would I have done? I would just all gone away defeated and lost my money (group 1, participant 2).

Moreover, a number of participants were confident about continuing with PA because of their positive experiences with Project MOVE; *“Now we know how to do the exercises, or what exercises to do”* (group 4, participant 4) *and “We could [now] create our own circuit”* (group 4, participant 2).

### 3.4 Motivation to MOVE

The majority of participants indicated that being part of Project MOVE, in general, was a motivating factor to start or continue being active. Specifically, participants reported that the $500 incentive motivated them to increase PA levels particularly because they didn't want to let their team members down, as explained by 1 participant; “*I think it did. It felt more like a team thing to me. Like I needed to show up… Like it was something that we were working together towards something at the end”* (group 1, participant 4).

Participants also reported that the positive health benefits they were beginning to experience motivated them to continue engaging in PA post‐intervention. One participant noticed her strength improved from 1 class to the next and commented, “*I can't believe how much better I've gotten, suddenly I was getting stronger”* (group 7, participant 3). Other participants also noticed improvements; “*My breathing and my endurance is better*” (group 7, participant 2) and “*we can recover so much faster*” (group 7, participant 1).

Some participants also suggested that goal setting was important in motivating them to be more active and remain active in the future, further recommending that the first Project MOVE session should be dedicated to setting achievable individual and group goals. As indicated by 1 participant*, “Having some sort of goals at the start I think would really help motivate us. And being able to say part way through, hey guys we're on track or we're not”* (group 10, participant 2).

Several groups also reported that a list of community PA programs and additional educational resources specific to PA and BC would further motivate them to get more involved and try different things. Consistent with many responses, one participant explained; “*I think a list of resources would be really helpful… here's where you can go to rent a bike or rent a paddleboard”* (group 10, participant 2). Additionally, participants recommended that health professionals (eg, dieticians, physiotherapists) could be invited to speak at one of the sessions as they valued learning about how to continue to make healthy lifestyle choices from credible professionals.

## 4 DISCUSSION

Consistent with models of post‐traumatic growth and PA,^14^, ^31^ one of the key findings was that Project MOVE offered an opportunity for women to be active with “similar others” and this fostered social support. By participating in community‐based initiatives among “similar others,” it has been suggested that BC survivors build autonomy and confidence in their ability to perform PA.^14^, ^15^, ^16^ Importantly, Project MOVE reframed the traditional support groups commonly offered to BC survivors to focus more on PA and movement, and this fostered positive emotions, enjoyment, and motivation.^14^ This dialog holds potential for changing the narrative from physical limitations and what the body can no longer do to a positive and inspirational one, empowering women to move and overcome their limitations. Drawing on self‐efficacy theory,^32^ it may be that having role models and these vicarious experiences with other women within Project MOVE was foundational to building confidence in PA that led to improved strength, fitness, and overall wellbeing. These outcomes are likely to build sustainability for PA participation.

The acceptability of the microgrants component of the program is also supported by the findings. From a theoretical perspective, many behavior change models highlight the importance of autonomy and behavioral control as key factors in PA participation (self‐determination theory,^33^ self‐efficacy theory,^32^ theory of planned behavior^34^). By allowing survivors the autonomy to define their own PA programs, the Project MOVE model helped to deconstruct contemporary views of what a PA program entails and demonstrated new contexts under which PA can be performed. Furthermore, the leader was an important factor in the acceptability of Project MOVE. Women in groups who had engaged and involved leaders reported more accountability to their group, which may have fostered group cohesion that was associated with higher enjoyment and participation. These findings mimic common outcomes in sport and exercise literature.^35^, ^36^ Based on these findings, microgrant models may be enhanced by providing leadership training and highlighting common strategies to build group cohesion (eg, group member roles, group norms, group goals).

Unique to Project MOVE was the addition of a $500 financial incentive for groups that increased their mean PA at 6 months follow‐up. The use of financial incentives among adults has been found to have a significant positive effect on PA session attendance, adherence, and maintenance over a 6 month period.^26^, ^37^ The financial incentives in Project MOVE acted to motivate participants to increase their activity and provided them with a sense of accountability to attend each PA session. While not discussed in the focus groups, 1 caution to this financial incentive may be a fostering of introjected regulation, which is a controlling form of motivation that could hinder longer‐term engagement.^38^ As such, the financial incentive may have a functional timing that needs to be limited. Nonetheless, introjected regulation may be an important form of motivation to get women started in their PA pursuits.^39^ In the duration and timing of Project MOVE, there were more benefits to the financial incentive in providing reinforcement for achievement.

### 4.1 Study limitations

The views presented reflect a specific population and thus cannot be generalized across the many diverse populations and settings across Canada or elsewhere. For instance, this study promotes a group setting; however, there may be BC survivors and other individuals who would prefer to be active independently. Also, some individuals and/or groups of individuals may find it easier to engage in PA if it is structured and prescribed, and thus are not interested in developing their own initiatives but rather would prefer to just “show up” and be told what to do. In addition, this study was not a randomized control trial and thus the true effect of the Project MOVE intervention on PA behaviour is not known. Future experimental research (ie, randomized control trial) comparing the various intervention components would provide a greater insight regarding the impact of Project MOVE on PA behaviour change. Finally, focus groups were used to collect information and may have limited individual perceptions of Project MOVE.

### 4.2 Clinical implications

The Project MOVE model (microgrants + financial incentives) shows promise as a strategy for initiating and maintaining PA engagement within the BC population. Most importantly, this model provided a starting point for survivors to overcome some of the barriers they often face (eg, lack of self‐confidence, financial constraints). As such, participants have been provided with the education and tools they need to self‐manage and sustain their future PA. Moreover, with the funds received, participants were able to try new activities at many community facilities and with a number of health and fitness professionals. The community partnerships that developed during the sessions may aid in further sustainability as participants are now more confident to take advantage of the many centres throughout the community. In turn, many of these facilities and professionals are more aware of the unique needs of this population and could offer more cost‐effective programs that are of interest to this population. In addition, Project MOVE was primarily focused on supporting engagement in PA organically; thus, few resources were provided, and little time was spent discussing other health behaviors. The absence of these was identified by many participants and thus needs to be considered in refining this model for wider dissemination.

### 4.3 Conclusion

Prioritizing Project MOVE participant views is an important first step in determining the feasibility of this novel program for long‐term initiation. Project MOVE provided a positive and autonomous environment for participants and enabled them to overcome many of the barriers to PA. This innovation shows great promise for increasing PA among BC survivors.

## FUNDING

This work was supported by the Lotte and John Hecht Memorial Foundation Innovation Grant of the Canadian Cancer Society (grant #702913).

## ETHICS APPROVAL

Ethical approval was obtained from the Behavioral Research Ethics Board at the University of British Columbia (#H14‐02502).

## CONFLICT OF INTEREST

None declared.

## Supporting information


**Appendix 1.** Flow summary of the Project MOVE protocol

**Appendix 2.** Focus group questions

**Appendix 3.** Characteristics of participants who completed the program evaluation questionnaire (*n* = 72) and participated in the focus groups (*n* = 52).
