# Supplementary material for: Acceptability and satisfaction of project MOVE: A pragmatic feasibility trial aimed at increasing physical activity in female breast cancer survivors
Source: Psychooncology. 2018 Mar 1;27(4):1251–6. doi: 10.1002/pon.4662 (PMC5947748; doi:10.1002/pon.4662)
Supplement: Supplementary file 1 — Appendix 1. Flow summary of the Project MOVE protocol [file PON-27-1251-s001.docx]

**Appendix 1**. Flow Summary of the Project MOVE Protocol

Project MOVE microgrant applications open

Advertising and Recruitment

Round 1 submissions:

-11 received

-5 funded

Microgrant applications close, applications reviewed

Funding recommendations and distribution of funds

Round 2 submissions:

-7 received

-5 funded

Baseline data collection (10 groups, n=87)

Project MOVE programs/initiatives begin

Reasons for drop-out:

-deterioration of health (n=9)

-could not be reached (n=2)

-not interested (n=2)

-death (n=2)

6-month follow-up data collection (n=72)

Program evaluation questionnaire (n=72)

Focus groups (10 groups, n=52)

Distribution of $500 financial incentive

↓
