# Supplementary material for: Acceptability and satisfaction of project MOVE: A pragmatic feasibility trial aimed at increasing physical activity in female breast cancer survivors
Source: Psychooncology. 2018 Mar 1;27(4):1251–6. doi: 10.1002/pon.4662 (PMC5947748; doi:10.1002/pon.4662)
Supplement: Supplementary file 2 — Appendix 2. Focus group questions [file PON-27-1251-s002.docx]

**Appendix 2.** Focus group questions

| 1. What words come to mind when you think about your experience in Project MOVE? |
| --- |
| 1. If you could provide future Project MOVE groups just starting up with some ideas and advice about planning a physical activity program for women affected by breast cancer, what would you tell them? |
| 3. What would you tell a new Project MOVE group about the challenges or barriers that  might be experienced by women affected by breast cancer that could affect their  participation? |
| 1. What advice do you have for a new Project MOVE group about how they might design their program to help women overcome these challenges or barriers? |
| 1. Based on your experience as a participant in Project MOVE – do you have any overall advice for group leaders who want to plan or organise a group like this for women affected by breast cancer. |
| 1. How important do you think the Project MOVE model is for enabling women affected by breast cancer to engage in physical activity? |
| 1. Do you have any final suggestions or recommendations on ways we can improve Project MOVE? |
| 1. If you had an opportunity to provide a brief message to another breast cancer survivor about physical activity in general, what would it be? |
