# Supplementary material for: Acceptability and satisfaction of project MOVE: A pragmatic feasibility trial aimed at increasing physical activity in female breast cancer survivors
Source: Psychooncology. 2018 Mar 1;27(4):1251–6. doi: 10.1002/pon.4662 (PMC5947748; doi:10.1002/pon.4662)
Supplement: Supplementary file 3 — Appendix 3. Characteristics of participants who completed the program evaluation questionnaire (n = 72) and participated in the focus groups (n = 52). [file PON-27-1251-s003.docx]

**Appendix 3.** Characteristics of participants who completed the program evaluation questionnaire (n=72) and participated in the focus groups (n=52).

| Variable | Completed program evaluation  (n=72) | Participated in focus groups  (n=52) |
| --- | --- | --- |
| Mean Age (years±SD) | 58.5±8.8 | 58± 8.76 |
| Age (years) % (n) |  |  |
| 35-44 | 5.6 (4) | 5.8(3) |
| 45-54 | 20.8 (18) | 28.9 (15) |
| 55-64 | 38.9 (29) | 38.5 (20) |
| 65-74 | 23.6 (17) | 21.2 (11) |
| 75-84 | 1.2 (1) | 0 (0) |
| Missing | 4.2 (3) | 5.8 (3) |
| Body Mass Index (kg/m^2^±SD) | 25.7±5.0 | 25.5±5.1 |
| Ethnicity: % (n) |  |  |
| White | 94.9 (68) | 96 (50) |
| Asian | 4.2 (3) | 2 (1) |
| Black | 1.4 (1) | 2 (1) |
| Education: % (n) |  |  |
| College or technical diploma or certificate | 36.1 (26) | 38.5 (20) |
| University Degree | 29.2 (21) | 25 (13) |
| Some post-secondary without diploma or degree | 18.1 (13) | 19.2 (10) |
| High school diploma | 9.7 (7) | 9.6 (5) |
| Some high school or less | 1.4 (1) | 0 (0) |
| Other | 5.6 (4) | 7.7 (4) |
| Martial Status: % (n) |  |  |
| Married or living with a life partner | 68.1 (49) | 69.2 (36) |
| Living alone | 26.4 (19) | 26.9 (14) |
| Widowed | 5.6 (4) | 3.8 (2) |
| Employment: % (n) |  |  |
| Retired | 33.3 (24) | 25 (13) |
| Full time work | 29.2 (21) | 38.5 (20) |
| Part time work | 16.7 (12) | 19.2 (10) |
| Recovering from illness/disability | 8.3 (6) | 9.6 (5) |
| Caring for family/managing household | 4.2 (3) | 1.9 (1) |
| Unemployed | 2.8 (2) | 1.9 (1) |
| Other | 5.6 (4) | 3.8 (2) |
| BC staging: % (n) |  |  |
| Stage 0 | 8.3 (6) | 9.6 (5) |
| Stage I | 15.3 (11) | 11.5 (6) |
| Stage II | 23.6 (17) | 21.2 (11) |
| Stage III | 12.5 (9) | 17.3 (9) |
| Stage IV | 5.6 (4) | 3.8 (2) |
| Unknown | 13.9 (10) | 15.4 (8) |
| No breast cancer | 20.8 (15) | 21.2 (11) |
| BC treatment † |  |  |
| Lymph or axillary node dissection | 65.3 (47) | 69.2 (36) |
| Radiotherapy | 55.6 (40) | 53.1 (28) |
| Chemotherapy | 50 (36) | 50 (26) |
| Lumpectomy | 45.8 (33) | 46.2 (24) |
| Reconstructive surgery | 29.2 (21) | 32.7 (17) |
| Hormonal Therapy | 27.8 (20) | 30.8 (16) |
| Single Mastectomy | 27.8 (20) | 32.7 (17) |
| Double Mastectomy | 19.4 (14) | 19.2 (10) |
| Other | 4.2 (3) | 3.8 (2) |
| Menopause Status |  |  |
| Pre-menopausal | 8.3 (6) | 9.6 (5) |
| Going through menopause | 12.5 (9) | 13.5 (7) |
| Post-menopausal | 62.5 (45) | 61.5 (32) |
| Missing | 16.7 (12) | 15.4 (8) |

† Participants were asked to indicate all that apply.
